# A rare case of tuberculous myocarditis: A diagnostic challenge in a tuberculosis‐endemic country

**DOI:** 10.1002/ccr3.8224

**Published:** 2023-11-16

**Authors:** Ghulam Abbas Shaikh, Samina Yaqoob, Fareeha Batool, Radeyah Waseem, Hussain Haider Shah, Asim Ali Abbasi, Nawaz Lashari, Tirth Dave

**Affiliations:** ^1^ Department of Cardiology Dr. Ruth K. M. Pfau, Civil Hospital Karachi Karachi Pakistan; ^2^ Dow University of Health Sciences Karachi Pakistan; ^3^ Bukovinian State Medical University Chernivtsi Ukraine

**Keywords:** diagnostic challenge, endemic country, myocarditis, tuberculosis, tuberculous myocarditis

## Abstract

**Key Clinical Message:**

Tuberculous myocarditis is a rare presentation of tuberculosis, posing diagnostic challenges in endemic countries. Clinicians should consider this entity in patients with unexplained heart failure, conduction abnormalities, or sudden cardiac events in tuberculosis‐endemic regions.

**Abstract:**

Tuberculous myocarditis is an uncommon manifestation of tuberculosis, often presenting as a diagnostic challenge, particularly in tuberculosis‐endemic regions. We report a case of a 58‐year‐old male with a history of chronic cough and fever, who presented with progressive dyspnea, generalized body swelling, and New York Heart Association (NYHA) Class IV heart failure. Clinical examination revealed signs of cardiac decompensation and congestive heart failure. Emergency echocardiography demonstrated biventricular dysfunction, and imaging showed clots in both atria and the left ventricle. The patient responded well to initial treatment with anticoagulants, antibiotics, diuretics, and inotropic support. Subsequent investigations, including computed tomography pulmonary angiogram (CTPA) and high‐resolution computed tomography (HRCT), confirmed active pulmonary tuberculosis. Anti‐tuberculous treatment (ATT) was initiated, and the patient showed remarkable improvement. The diagnosis of tuberculous myocarditis was based on clinical, radiological, and laboratory evidence, as cardiac biopsy was not performed due to resource limitations. Tuberculous myocarditis is an underreported condition, and clinicians should be vigilant about its occurrence, especially in tuberculosis‐endemic regions. Early recognition and prompt initiation of ATT can lead to favorable outcomes. This case highlights the importance of considering tuberculous myocarditis in patients with unexplained heart failure or cardiac abnormalities in areas with a high burden of tuberculosis.

## Graphical abstract

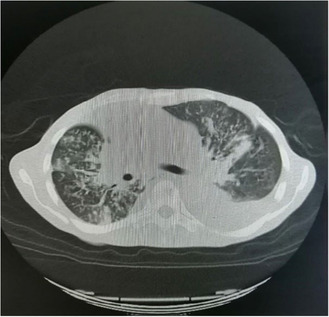

## 1 INTRODUCTION

Tuberculosis (TB) is still one of the deadliest diseases worldwide. TB affecting the myocardium is rare, with a prevalence of less than 0.2%, according to several studies.^1^, ^2^ Although tuberculous myocarditis is typically asymptomatic, it can cause acute symptoms such as heart failure, ventricular fibrillation, sudden cardiac arrest, long QT syndrome, and dilated cardiomyopathy.^3^ Pakistan is among the top five countries with the highest burden of TB cases in the world, with approximately 510,000 new cases reported every year and around 15,000 of these cases being drug resistant. The country accounts for 61% of the total TB cases in the WHO Eastern Mediterranean Region. Pakistan also has the fourth‐highest rate of multidrug‐resistant TB (MDR‐TB) globally.^4^ This article will discuss the clinical presentation, diagnostic workup, and treatment given to a 58‐year‐old male patient with tuberculous myocarditis.

## 2 CASE PRESENTATION

A 58‐year‐old male with no known comorbid conditions, ex‐smoker of 20 packs per year came to the Cardiac Emergency of Civil Hospital Karachi with complaints of Cough and fever for the one and a half months, progressive shortness of breath (SOB) and generalized body swelling for the past 15 days.

The patient was alright one and half months back when he started to experience a cough, which was productive, persistent and progressive in nature. Sputum was greenish in color not blood stained associated with febrile illness, which was low‐grade, undocumented and intermittent. For the last 15 days, the patient has been complaining of SOB progressive in nature (New York Heart Association NYHA Class IV) associated with orthopnea, paroxysmal nocturnal dyspnea and generalized body swelling.

Past medical history was insignificant. No significant drugs or transfusion history. Personal history is positive for undocumented significant weight loss and decreased appetite.

On examination, he was sick looking middle‐aged male below average built, dyspneic, drowsy but oriented to time, place and person. He had a blood pressure of 70/50 mmHg, Pulse of 130 beats per minute low volume and regular, respiratory rate of 40 breaths per minute and oxygen saturation of 75% (Table 1). He was anemic, jaundiced, jugular venous pressure up to earlobe, edematous and cervical lymphadenopathy.

**TABLE 1:** Arterial blood gases.

Labs	Normal values	Results
pH (mmHg)	7.35–7.45	7.50
po_2_ (mmHg)	75–100	50
pco_2_ (mmHg)	35–45	25
HCO_3_ (mEq)	22–26	28
Saturated O_2_	> 95%	65%
Lactate (mmol/L)	< 2	4

abbreviations: hco_3_, bicarbonate; pco_2_, partial pressure of carbon dioxide; pH, potential hydrogen.

**FIGURE 1:**
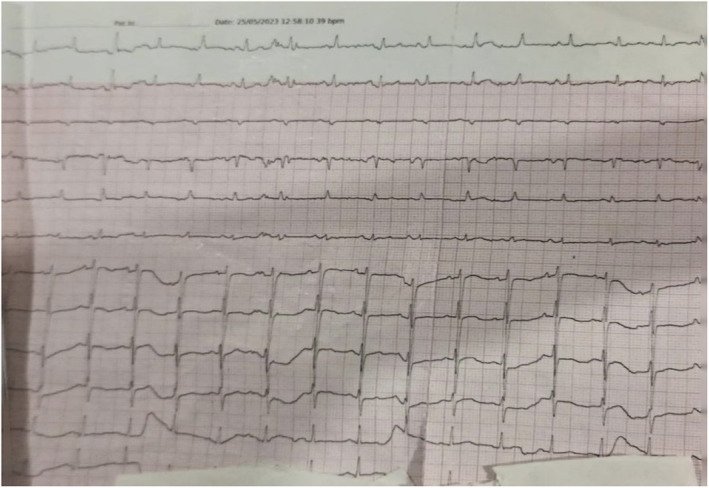
Emergency ECG showing sinus tachycardia. Arterial blood gases were also ordered which revealed the following picture.

On cardiovascular examination, the apex beat was at the sixth Intercostal Space (ICS) lateral to the left mid clavicular line (MCL), sustained and heaving in character. Right parasternal heave present. The first heart sound audible with second heart sound splits on inspiration along with S3 gallop. Bilateral coarse crepitations are present mostly on the right upper and middle lobe. All baseline investigations were sent emergency electrocardiogram revealed sinus tachycardia with no significant ST‐T changes (Figure 1).

**FIGURE 2:**
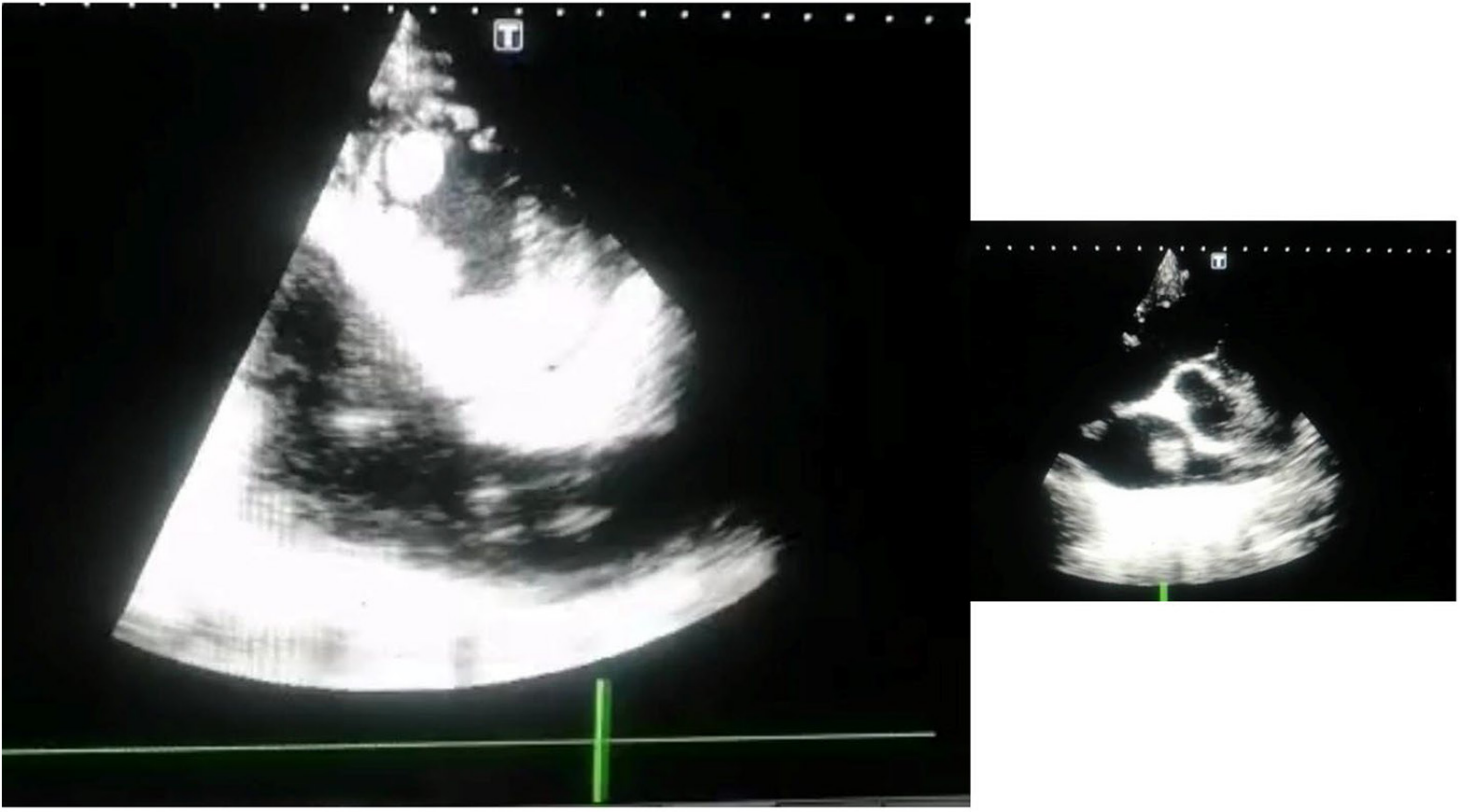
Echo diagram.

**FIGURE 3:**
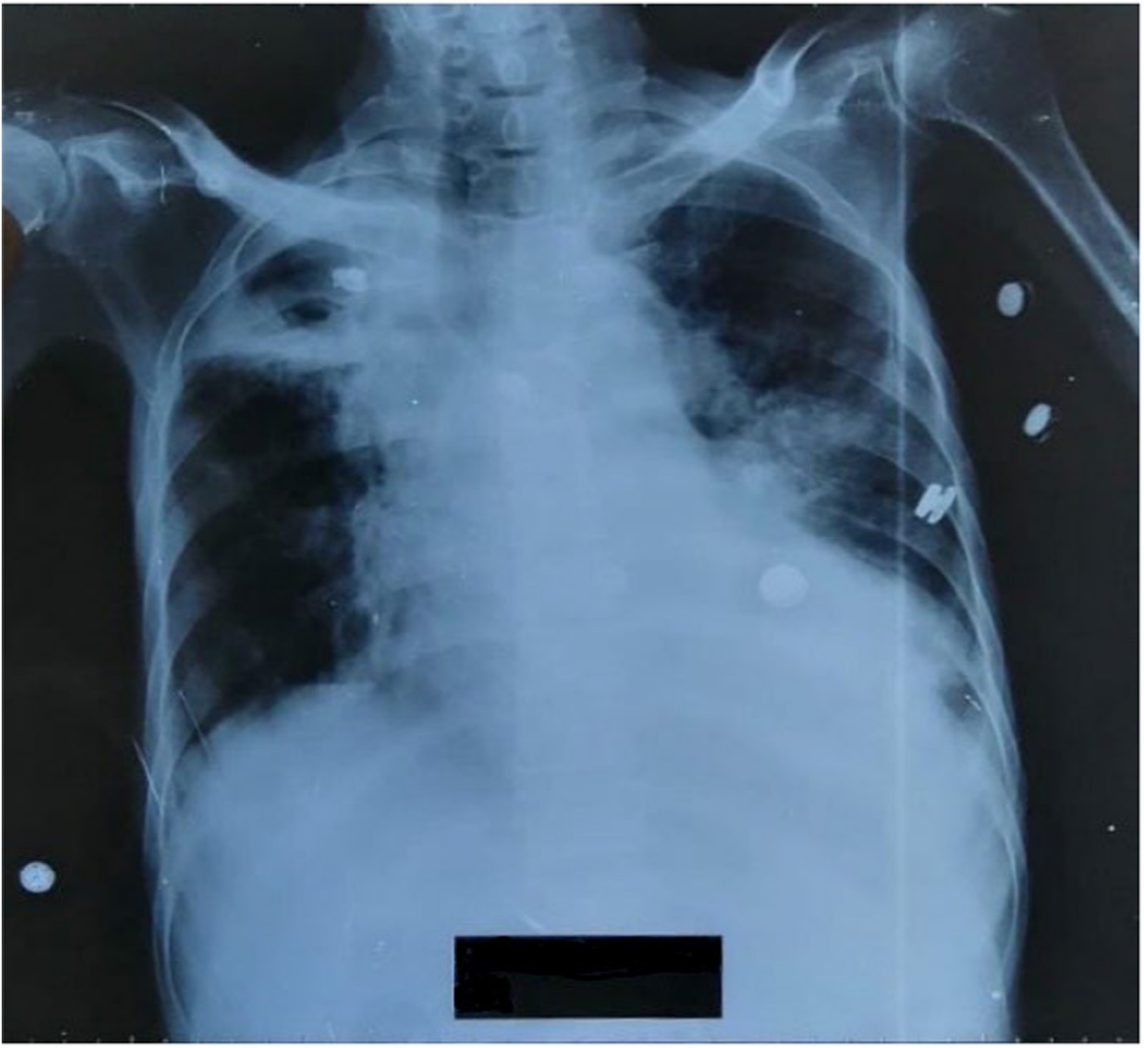
Chest x‐ray.

After reviewing the ABG reports the patient was started on 5 L O_2_ via nasal cannula. Ionotropic support of 0.05 μg/kg/min noradrenaline was started. Immediate bedside echocardiography was done which revealed biventricular dysfunction with preserved left ventricular (LV) dimensions, left ventricular ejection fraction (LVEF) of 20%–25% and tricuspid annular planer systolic excursion (TAPSE) of 08 mm along with multiple clots in right atrium (RA) and LV apex. He also had thickened mitral valve, moderate mitral regurgitation (MR) and mild aortic regurgitation (AR) (Figure 2). Portable chest X‐ray was also done which non homogenous opacity on right upper zones along with bilateral pleural effusions (Figure 3). On the basic of these reports (Table 2), the patient was started on antibiotics, digoxin, intravenous (IV) furosemide and therapeutic anticoagulant. The patient was shifted to coronary care unit (CCU).

**TABLE 2:** Lab reports of the patient during the hospital stay.

Parameters	Reference values	On admission
Hematologic tests and coagulation profile		
Hb (g/dL)	13.5–17.5 (male)/12.0–15.5 (female)	10.2
HCT (%)	38.8–50.0 (male)/34.9–44.5 (female)	34.2
MCV (fL)	80–96	76.1
MCH (Pg)	27–31	23.4
MCHC (Gm/dL)	32–36	30.8
TLC (10^3^/μL)	4.0–11.0	7.1
Neutrophils (%)	40–75	73
Lymphocytes (%)	20–45	20
Platelets (10^3^/μL)	150–400	80
ESR (mm/h)	0–15	140
CRP (mg/L).	<0.3	44.5
LDH (units/L)	140 to 280	850
Troponin I	0 and 0.04 ng/mL	10
PT (s)	11–13	11.7
INR	0.8–1.2	1.5
APTT (s)	28–40	
Electrolytes		
BUN (mg/dL)	7–20	22
Creatinine (mg/dL)	0.6–1.3	0.5
Na^+^(mEq/L)	135–145	126
K^+^ (mEq/L)	3.5–5.0	4.4
Cl^−^ (mEq/L)	95–105	99
Ca_2_^+^ (mg/dL)	8.5–10.5	8.4
Mg_2_^+^ (mg/dL)	1.7–2.4	1.9
Phosphorus (mg/dL)	2.5–4.5	2.7
Liver function tests		
Total bilirubin (mg/dL)	0.1–1.2	2.7
SGPT (U/L)	0–40	44
ALP (U/L)	40–130	247
Protein A/G ratio		
Total protein (G/dL)	6.0–8.3	6.4
Albumin (G/dL)	3.5–5.0	2.3
Globulin (G/dL)	2.3–3.5	4.1
TSH (mIU/L)	0.5 to 5.0	0.685

Abbreviations: A/G, albumin/globulin; ALP, alkaline phosphatase; aPTT, activated partial thromboplastin time; BUN, blood urea nitrogen, CA, calcium; CRP, C‐reactive protein; ESR, erythrocyte sedimentation rate; hb, hemoglobin; HCT, hematocrit; INR, international normalized ratio; K, potassium chloride; LDH, lactate dehydrogenase; MCH, mean corpuscular hemoglobin; MCHC, mean corpuscular hemoglobin concentration; MCV, mean corpuscular volume; Mg, magnesium; NA, sodium; PT, prothrombin time; RBC, red blood cell; SGPT, serum glutamic pyruvic transaminase; TLC, total leucocyte count.

**FIGURE 4:**
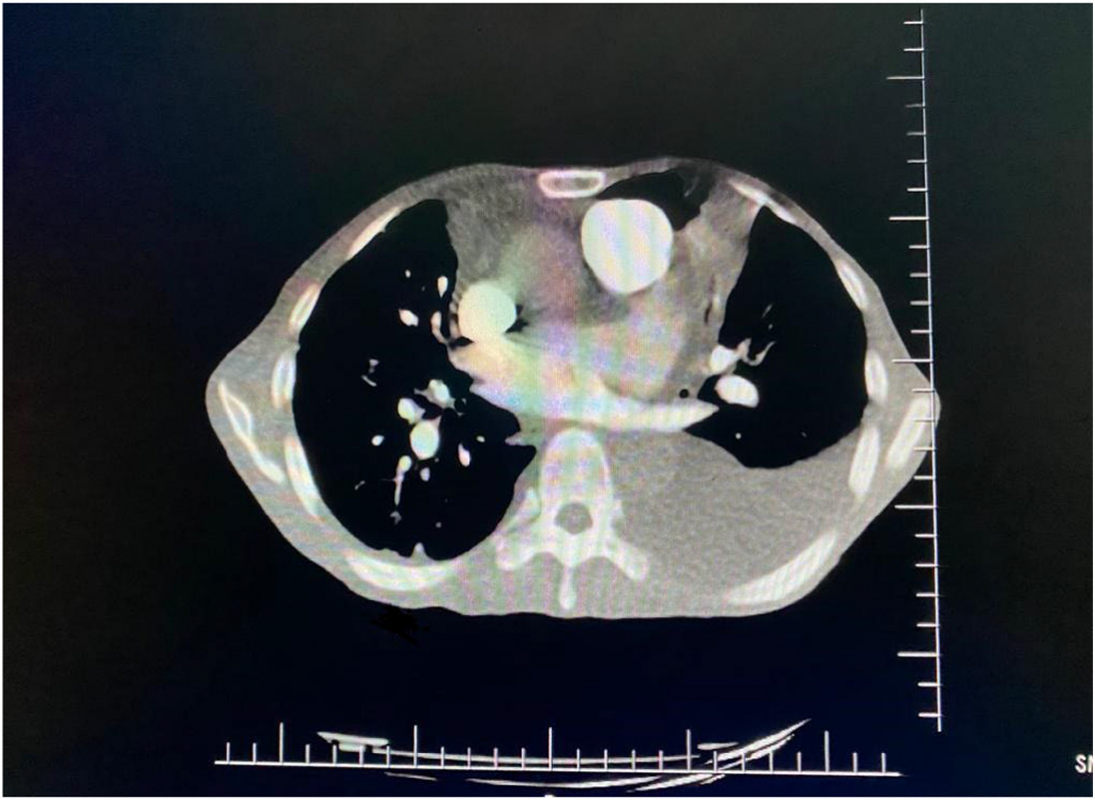
Computed tomography pulmonary angiogram (CTPA).

After 24 h, the patient improved on anticoagulants, antibiotics, and diuretics. Inotropic support was stopped. He was started on low‐dose heart failure treatment. The patient improved drastically and came out of heart failure after 72 h. To rule our cause of biventricular heart failure, left heart catheterization was done which was normal. On suspicion of pulmonary embolism (PE) and primary lung pathology, CTPA (Figure 4), and high resolution computed tomography (HRCT) were done which showed filling defect in sub segmental branches of right main pulmonary artery and this leads to small wedge shaped patch of consolidation.

**FIGURE 5:**
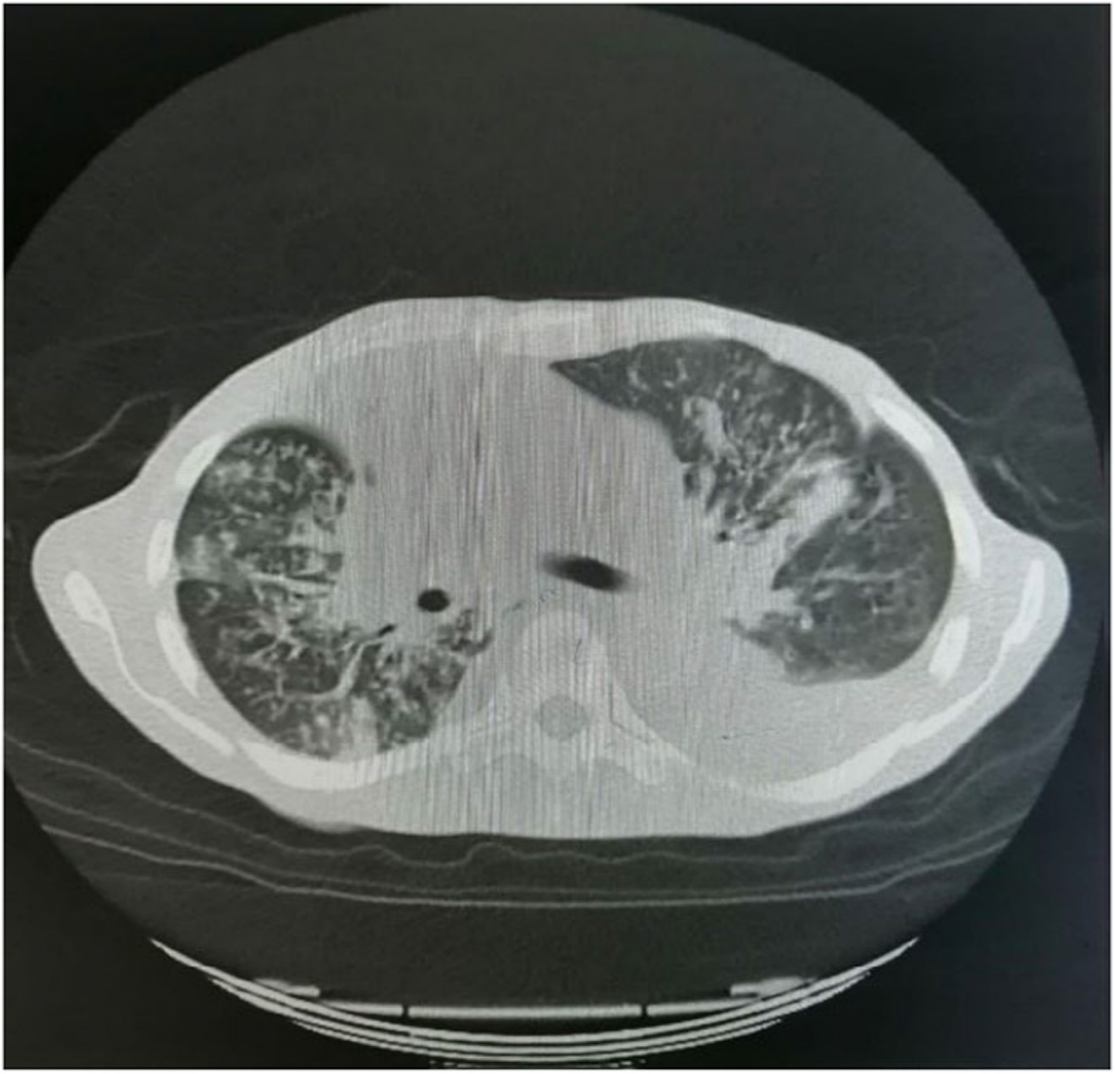
High resolution computed tomography (HRCT).

HRCT (Figure 5) were ordered which revealed bilateral pleural effusions mild on the right and gross on the left side with collapse and consolidation of adjacent parenchyma. Multifocal areas of nodular infiltrates giving tree on bud configuration are diffusely scattered in bilateral lung parenchyma more marked on right side. These findings are consistent with active TB.

After these reports the infectious diseases (ID) Department was consulted. They started anti‐tuberculous treatment (ATT) including rifampicin, isoniazid (plus Vitamin B6), pyrazinamide, and ethambutol and shifted the patient to isolation in negative pressure room. Three early morning sputum samples for TB testing were sent, which confirmed a positive result. It has been determined that TB is present. Prednisolone was also given because of suspected TB myocarditis. To rule out other infectious etiology hepatitis B, hepatitis C and an anti‐HIV antibody test were done where were all non‐reactive.

**FIGURE 6:**
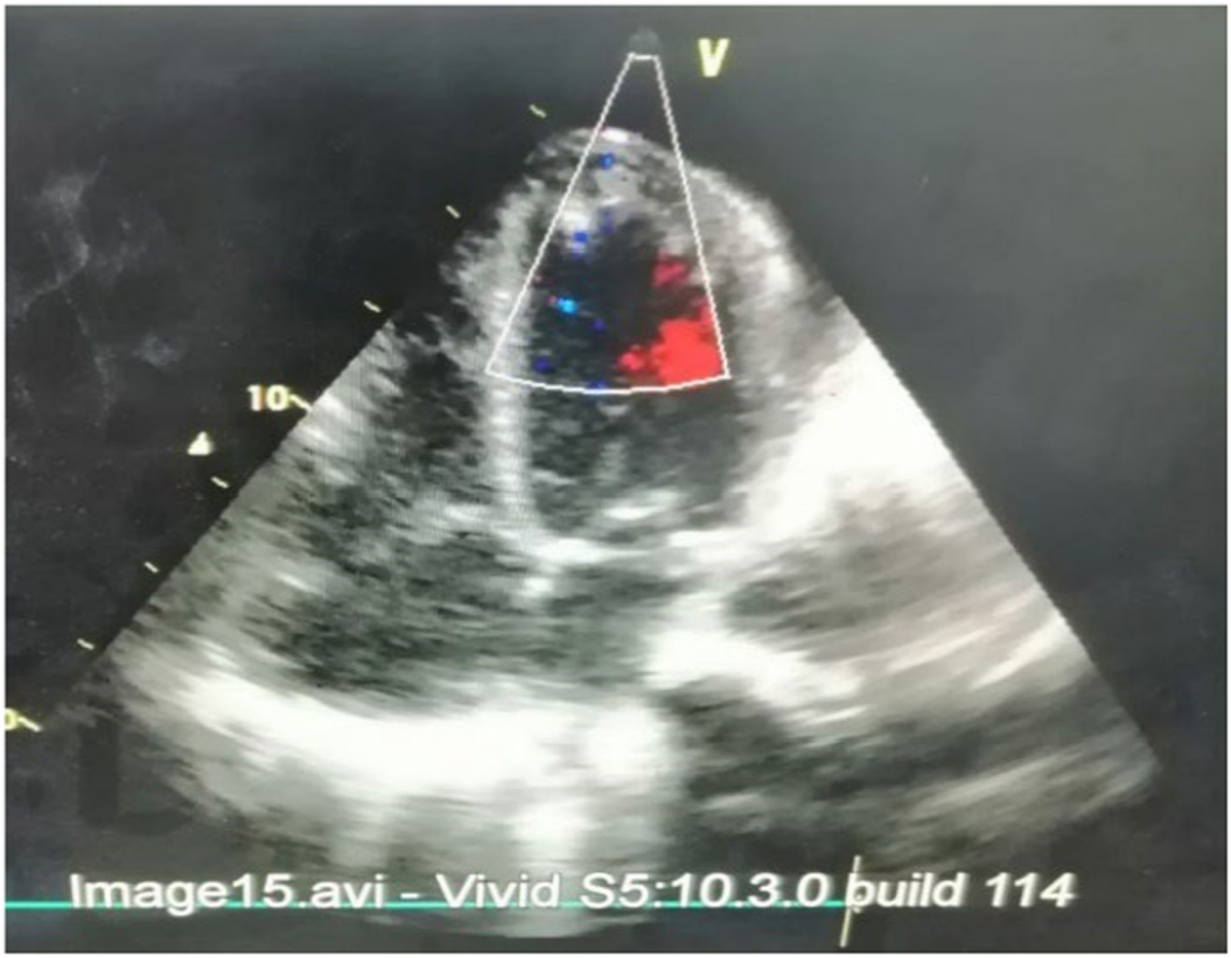
Second echo.

Repeated echocardiography was done after 2 weeks of presentation which showed complete resolution of only right atrial (RA) clots with reduction in the size of left ventricular clots (Figure 6).

Patient was discharged on heart failure, anticoagulants and ATT drugs with a diagnosis of tuberculosis myocarditis and for a follow‐up in TB center. The diagnostic challenge was that tuberculosis myocarditis is definitely diagnosed on cardiac biopsy but due to the lack of resources, we diagnosed this patient on clinical and radiological grounds, and he improved on tuberculous medications. The patient did not attend the scheduled follow‐up appointment.

## 3 DISCUSSION

Cardiac involvement in TB is a rare finding accounting for only about 1% of patients.^2^ A literature search on PubMed yielded no reported cases of this condition in Pakistan, despite Pakistan being a tuberculosis‐endemic country, accounting for 5.7% of the Global Tuberculosis Burden in 2020.^4^, ^5^ It is alarming that cases of tuberculous myocarditis have not been previously reported in the country, which may be due to the various non‐specific clinical findings such as congestive heart failure, conduction disorders, myocardial infarction, and sudden cardiac death and the diagnostic difficulty it imposes.^6^

Myocardial infiltration by TB may result from direct extension of pericardial lesions, lymphatic spread from mediastinal lymph nodes, or via the blood from a remote focus.^7^ Three varieties of myocarditis have been reported based on the underlying histology: caseating nodular, miliary, and diffuse infiltrative.^8^ The discrimination between these subtypes remains unspecified in our patient, as a biopsy was not conducted.

The involvement of different layers of the heart has been reported in the literature with varying occurrence rates. Lopez et al. conducted a study on the cases reported between 1955 and 2020 and concluded that pericarditis accounted for 2%–5% of all cases reported for cardiac involvement in TB, followed by myocarditis in 0.14%–2% of cases and aortitis in 0.3% of the total cases.^9^ Another systematic review of cases conducted by Michira et al. reported that immunocompetent patients below the age of 45 bore the brunt of the disease, accounting for 81% of the cases. A slightly higher predominance of males compared to females was also founded (2:1 ratio).^3^ This is in discordance with our case, where the patient is 58 years old, again exemplifying the rarity of this presentation.

The ubiquitous presentation of the condition poses a challenging diagnosis, causing most cases to be identified at autopsy.^10^ Endomyocardial biopsy may also yield a diagnosis; however, it is limited due to the entailing complications and procedural limitations. The diagnosis constitutes a detailed medical history, clinical presentation, supportive imaging, and laboratory findings. Clinical diagnosis of myocarditis is warranted based on elevated cardiac markers such as Troponin I or T, creatinine kinase‐MB, and reduced left ventricular function proved by echocardiographic or cardiac MR findings.^6^ Our patient presented with the typical clinical findings and laboratory parameters, which, coupled with positive TB tests, confirmed the diagnosis.

Myocardial tuberculosis is always suspected in a diagnosed TB patient presenting with conduction defects, sudden cardiac death, ventricular and pseudo‐aneurysms, aortic insufficiency or coronary arteritis. These conditions, culminating in acute fulminant myocarditis, have been reported in two other cases, one presenting in a 30‐year‐old male and the other in a 32‐year‐old female.^11^, ^12^

In accordance with the 2013 European Society of Cardiology Working Group on Myocardial and Pericardial Disease guidelines, treatment is specific to the causative organism and includes administration of immunosuppressive or immunomodulatory therapies.^13^ In lieu of the guidelines, we initiated anti‐tuberculous therapy (ATT) comprising rifampicin, isoniazid, pyrazinamide and ethambutol, which concurrent administration of furosemide, metoprolol succinate, ramipril, and spironolactone.

Eosinophilic myocarditis is another related condition in patients already undertaking ATT as a side effect of Isoniazid, rifampicin, and pyrazinamide. Typical features include a rash, multi‐organ failure, and eosinophilia on peripheral blood film that resolves with the withdrawal of the causal drug.^14^ Idiopathic giant cell myocarditis is another similar condition typically affecting middle‐aged individuals with a histological picture of cardiomyocyte damage, granulomatous lesions, and eosinophils. The condition has a poor prognosis and is recalcitrant to tuberculous therapy.^11^

## 4 CONCLUSION

We present a rare case of tuberculous myocarditis presenting in a 58‐year‐old male diagnosed on clinical and laboratory investigations. Knowledge of the condition's signs, symptoms, and prevalence is vital for clinicians in Pakistan and other TB‐endemic regions. Tuberculous myocarditis follows a favorable prognosis, and early detection of the condition in vulnerable regions may spare patients from prolonged treatment complications and expenses.

## AUTHOR CONTRIBUTIONS

**Ghulam Abbas Shaikh:** Conceptualization; resources; writing – original draft; writing – review and editing. **Samina Yaqoob:** Writing – original draft; writing – review and editing. **Fareeha Batool:** Writing – original draft; writing – review and editing. **Radeyah Waseem:** Writing – original draft; writing – review and editing. **Hussain Haider Shah:** Writing – original draft; writing – review and editing. **Asim Ali Abbasi:** Writing – original draft; writing – review and editing. **Nawaz Lashari:** Writing – original draft; writing – review and editing. **Tirth Dave:** Software; supervision; writing – original draft; writing – review and editing.

## FUNDING INFORMATION

None.

## CONFLICT OF INTEREST STATEMENT

The authors have no conflict of interest to declare.

## ETHICS STATEMENT

The ethical approval was not required for the case report as per the country's guidelines.

## CONSENT

Written informed consent was obtained from the patient to publish this report.

## Data Availability

The data that support the findings of this article are available from the corresponding author upon reasonable request.
